# SIPA1 Regulates LINC01615 to Promote Metastasis in Triple-Negative Breast Cancer

**DOI:** 10.3390/cancers14194815

**Published:** 2022-10-01

**Authors:** Yuan Xiang, Lingyun Feng, Hui Liu, Yuhuan Liu, Jiapeng Li, Li Su, Xinghua Liao

**Affiliations:** 1 Department of Medical Laboratory, Central Hospital of Wuhan, Tongji Medical College, Huazhong University of Science and Technology, Wuhan 430014, China; 2 Key Laboratory of Molecular Biophysics of Ministry of Education, College of Life Science and Technology, Huazhong University of Science and Technology, Wuhan 430081, China; 3 Institute of Biology and Medicine, College of Life Sciences and Health, Wuhan University of Science and Technology, Wuhan 430081, China

**Keywords:** SIPA1, LINC01615, MMP9, breast cancer

## Abstract

**Simple Summary:**

Breast cancer is a malignant tumor that often endangers women. After undergoing surgery and supplementary chemotherapy, however, tumor recurrence has not been well researched. The primary cause is high metastatic rates. Hence, bioinformatic and functional analyses were performed to indicate the effect of LINC01615 on breast cancer. We revealed that LINC01615 is regulated by the transcription factor SIPA1 in promoting breast cancer cell malignancy.

**Abstract:**

Long non-coding RNAs (lncRNAs) are reported to play an important regulatory effect in carcinogenesis and malignancy. We found by high-throughput sequencing that LINC01615 is upregulated in breast cancer patients and reduces patients’ overall survival. In vivo and in vitro experiments, we clarified that overexpression of LINC01615 can promote breast cancer cell metastasis ability. The expression of LINC01615 is regulated by the transcriptional activator SIPA1, thereby promoting carcinogenesis in breast cancer cells. Our research clarified that LINC01615 can act as an oncogenic factor in promoting the development of breast cancer.

## 1. Introduction

Breast cancer is the commonest tumor type and the main cause of death in female cancer patients in female cancer patients worldwide [1,2], which has overtaken lung cancer for the first time, reported with 2.26 million cases in 2020 as the most common cancer worldwide (with 2.2 million cases of lung cancer) [3,4,5]. According to statistics, the cases of breast cancer in Europe, Asia, South America, and Africa are increasing year by year [1]. As a malignant tumor, breast cancer has been increasingly threatening women’s health in China in recent years. According to the statistics from the National Cancer Center of China, approximately 278,900 new breast cancer incidences in Chinese women in 2014 led to 16.51% of the incidence of female malignant tumors, which became the most common malignant tumors in females [6,7]. Although there have been significant advances in breast cancer therapy with the advancement of modern medicine, it remains deviation in diagnosis and failure in treatment, making it particularly important to explore the nosogenesis of breast cancer.

LncRNA (long non-coding RNA) is one of the non-coding RNAs with more than 200 nucleotides and cannot encode peptides [8,9]. LncRNAs play important roles in organisms, including regulating oncogenes in translational, inactive in sex-chromosome and alternative splicing. LncRNAs perform these functions through a variety of different mechanisms, including acting as molecular scaffolders, “guiding” chromatin modifying enzymes such as HOTAIR and DLX6AS, acting as competing endogenous RNAs (ceRNAs), and acting as “sponges” to adsorb miRNAs or proteins [8,10]. They can also promote or inhibit a wide range of chromatin interactions (e.g., LUNAR1 and CCAT1) and even act through their own transcriptional behavior [11]. Other mechanisms are also being investigated, such as the coordination between lncrnas and nuclear structures, the formation of circular lncRNAs, as well as lncRNA-induced mRNA disorder. LncRNAs have become key regulators of cancer metastasis pathways and biomarkers of related diseases. Increasing founding indicated that dysregulated lncRNAs involve in cancer cell proliferation, cancer progression, and cancer metastasis [10,12]. In breast cancer, a variety of non-coding RNAs have been found to work as tumor suppressors or tumor-promoting effectors, such as XIST, HOTAIR, GAS5, MALAT1, etc. [13,14,15,16]. These studies indicate that the maladjusted lncRNAs caused clinicopathologic malignancy and poor prognosis in breast cancer patients.

SIPA1 (signal-induced proliferation-associated 1) protein, a member of the RapGAP protein family, is a mitogen-induced GTPase containing 1042 amino acids [17]. SIPA1 protein is highly expressed in the spleen, bone marrow, thymus, and other lymphatic hematopoietic systems. Its first discovered function is as GTPase-activating protein (GAP) of RAS-related mediating protein Rap1. Catalyze the conversion of Rap1 from active form RAP1-GTP to inactivated form RAP1-GDP. SIPA1 protein showed specific GAP activity to RAS-related proteins (Rap1 and Rap2) [18,19,20] to inactivate the Rap protein from GTP binding to GDP binding and thus regulate cell proliferation and migration and other biological processes [21]. In mice, SIPA1 protein is mainly localized to the nucleus, and its highest expression is in the lymphatic hematopoietic system, such as the spleen, bone marrow, and thymus. Most studies focused on breast cancer with SIPA1 due to its migration and invasion promotion. Meanwhile, SIPA1 expresses the nuclear region, which can also be a predictor of lymph node metastatic status [22,23]. Previous studies confirmed that upregulated SIPA1 in breast cancer cell lines, SIPA1 activates the promoter activity of ITGB1 through transcription, which improves the adhesion ability of breast cancer cells, thereby improving the malignancy of breast cancer cells [23]. Further cell function experiments proved that knockdown of SIPA1 expression could reduce the adhesion, migration, and invasion of breast cancer cells. SIPA1 knocked down in breast cancer cells significantly reduced FAK and Akt phosphorylation and inhibited MMP9 extracellular secretion through the integrin-mediated FAK/AKT-MMP9 signaling pathway. Therefore, the mechanism causing breast cancer cell adhesion, migration, and invasion can be explained by SIPA1, which may be achieved by regulating the ITGB1/FAK/Akt-MMP9 signaling pathway. Current research has also found that SIPA1 protein can prove breast cancer cell stemness by targeting the CD44 gene, and enhance aerobic glycolysis of cancer cells by targeting the EPAS1 gene to promote cancer cell metastasis [21,24]. However, other ways of regulating the metastasis of breast cancer cells by SIPA1 remain to be explored [21,25].

In this study, we found a significant correlation between SIPA1-regulated lncRNA and tumor metastasis. The key SIPA1-regulated lncRNA molecule LINC01615 was screeded out from lncRNA sequencing data mining. Decreasing the expression of LINC01615 was used to decrease the migration and invasion in breast cancer cells. The results reveal a new mechanism that SIPA1 regulates breast cancer cell EMT and then promotes metastasis by up-regulating the expression of LINC01615, which provides a new way to clarify the process of metastasis in cancer.

## 2. Materials and Methods

### 2.1. Cell Culture

Human breast cancer cell lines BT549 and MDA-MB-231 were purchased from the Cell Bank of the Chinese Academy of Medical Science (Shanghai, China). The MDA-MB-231 cells were cultured with RPMI-1640 medium containing 10% FBS, and BT549 cells were cultured with DMEM medium containing 10% FBS. All cells were cultured in a 5% CO_2_ atmosphere at 37 °C.

### 2.2. Animal Experiments

Animal experiments were approved by the ethics committee of Wuhan University of Science and Technology. Containing 5 × 10^6^ cells, MDA-MB-231 in PBS were subcutaneously injected into 4-week-old female nude mice for 27. Then mice were euthanasia, and the in vivo tumor tissues were fixed with 4% paraformaldehyde at 4 °C for 48 h after excision and weighed. The tumor volume was measured by using a digital caliper and algorithm using the volume (mm^3^) = [width (mm)]^2^ × [length (mm)]/2. After fixation, tumor tissue slides were stained with hematoxylin and eosin (H&E) or used for immunohistochemistry.

### 2.3. Overexpression and Knockdown Cell Lines

Lentiviral particles were produced in HEK293T cells cotransfected with shSIPA1, shLINC01615, LV-LINC01615, or the corresponding empty vectors, and pMD2.G and psPAX2 plasmids to construct stabilized overexpression or knockdown cell lines. Then, 2 × 10^5^ MDA-MB-231 or BT549 cells were infected with 1 × 10^6^ lentivirus at 8 μg/mL puromycin.

### 2.4. RT-qPCR

Total RNA from cells and tissues was extracted with TRIzol reagent (Thermo Fisher Scientific, Waltham, MA, USA). Then, 1 μg RNA was reverse transcribed into cDNA using the HiScript III 1st Strand cDNA Synthesis Kit (Vazyme Biotech, Nanjing, China). Real-time quantitative PCR was conducted using Taq Pro Universal SYBR qPCR Master Mix (Vazyme Biotech, Nanjing, China) on a CFX96 Real-Time quantitative PCR Detection System (Bio-Rad Laboratories, Hercules, CA, USA). The primer sequences were as follows: LINC01615: 5′-GAAGACAGGGGATCCCGAAG-3′ and 5′-AATGAAAGTCCAGCAGGAGGG-3′; MIR100HG: 5′-CCCAGTGCAAGGACAAAGA-3′ and 5′-GCAGAGGAGGTGTCTTCAGG-3′; LINC00702: 5′-GCAGTGGCATGATCTCGGCT-3′ and 5′-GGCCGAGGCAGGTGGATAAC-3′; LINC02544: 5′-TCTCATTCGTGGCTGGATCA-3′ and 5′-ACGCTCTCGAAATCGTGTCC-3′; SERTAD4-AS1: 5′-CCTATTCCCTGCTTCTGCGA-3′ and 5′-AGCCAGAGGTCTGGTTTTTCA-3′; FENDRR: 5′-CCACATGGATGGTTGCCACTCTC-3′ and 5′-GCTGGTACTCGGCCTTCTAATTGG-3′; GAPDH: 5′-TGAACGGGAAGCTCACTGG -3′ and 5′-TCCACCACCCTGTTGCTGTA-3′.

### 2.5. Scratch-Wound Assays

The cells were inoculated in 6-well plates, and 12 h later, a 200 µL tip was used to gently scratch the cell surface, and then the scratches were washed with PBS. The scratches were photographed and analyzed under the microscope at 0, 12, and 24 h, respectively.

### 2.6. Transwell Assays

Briefly, 5 × 10^4^ cells were seeded in the upper chamber of Corning chambers, and the lower chamber was placed in a medium containing 10% FBS. After 24 h, the cells in the upper chamber were gently wiped off with a cotton swab, and the cells in the lower chamber were fixed with paraformaldehyde for 20 min, then stained with 0.1% crystal violet, and finally photographed and analyzed under a microscope.

### 2.7. Differential Expression Analysis

Using R language DESeq2 package in the filtered and then the lncRNA and mRNA expression level in the form of differential expression analysis, according to |log2FC| ≥ 2, q-value < 0.05 standard filtered set parameters.

### 2.8. Immunohistochemistry

For immunohistochemistry, the sections were incubated with the primary antibodies at 4 °C overnight and then with secondary antibodies at 25 °C for 1 h. The signals were visualized by a DAB kit and counterstained with hematoxylin.

### 2.9. Sequencing Data Filtering and Reference Genome Alignment

The resulting raw data are called raw reads. The raw data are first filtered, and the high-quality data after filtering are called clean reads. High-quality data were then compared with the reference genome and transcriptome.

### 2.10. Statistical Analysis

All experimental data were statistically analyzed and plotted using GraphPad Prism 8 software. The comparison between treatment and control groups was tested by unpaired Student’s *t*-test and Wilcox test, and the statistical results were the results of 3 or more independent replicates. For multiple comparisons, the one-way ANOVA plus two-sided Tukey test was applied. The two-way ANOVA was applied for the comparison of tumor volume data. All values are expressed as the mean ± SD unless otherwise indicated.

## 3. Results

### 3.1. SIPA1 Expression Is Elevated in Breast Cancer

**Figure 1:**
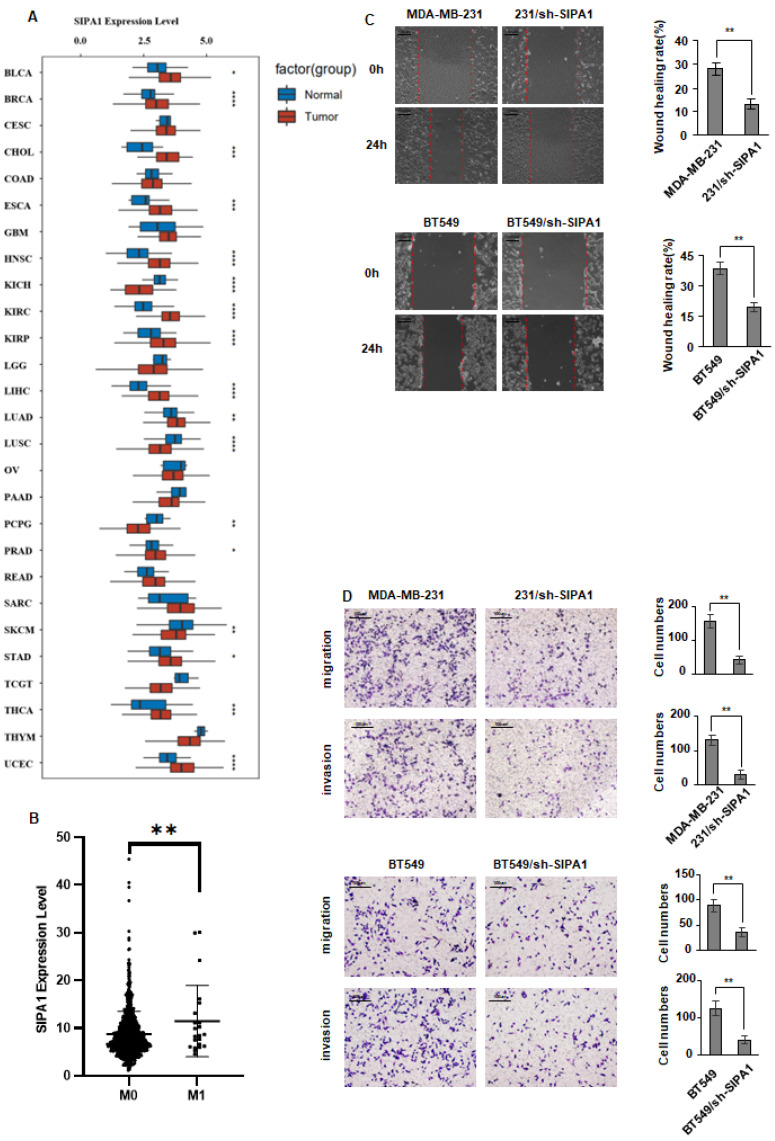
SIPA1 expression is elevated in breast cancer. (**A**) The expression of SIPA1 in various tumor tissues is different from that in normal tissues. (**B**) The difference in SIPA1 expression between breast carcinoma in situ and metastatic tissues. (**C**) Cell-wound-healing ability was inhibited in SIPA1 knockdown cells. (**D**) Chamber invasion ability was inhibited in SIPA1 knockdown cells. Data represent the means ± SD. ** *p* < 0.01.

To investigate the SIPA1prognostic valuable in generalized cancer, gene expression data in breast cancer tissues and normal breast tissues were obtained from the TCGA database. The results showed that *SIPA1* was significantly upregulated in breast cancer tissues. Indicating the occurrence and development of breast cancer could be blamed for overexpressed *SIPA1* (Figure 1A). To further verify the correlation between *SIPA1* and breast cancer metastasis, the gene expression data of breast cancer in situ tumor tissues (M0) and metastatic tumor tissues (M1) were obtained from the TCGA database, and their differential expression was analyzed. SIPA1 expression in metastatic tumor tissues was significantly upregulated than that in orthotopic tumor tissues, suggesting SIPA1 might be correlated with breast cancer metastasis (Figure 1B). To verify the biological function of SIPA1 in breast cancer cells, SIPA1 was knocked down in BT549 and MDA-MB-231 cells. The wound-healing and Transwell assay performed that the migration and invasion ability of cells was inhibited after SIPA1 knockdown (Figure 1C,D). These results suggest that SIPA1 may promote the occurrence and development of breast cancer by promoting the proliferation and migration of breast cancer cells.

### 3.2. SIPA1 Regulates LINC01615 Expression

**Figure 2:**
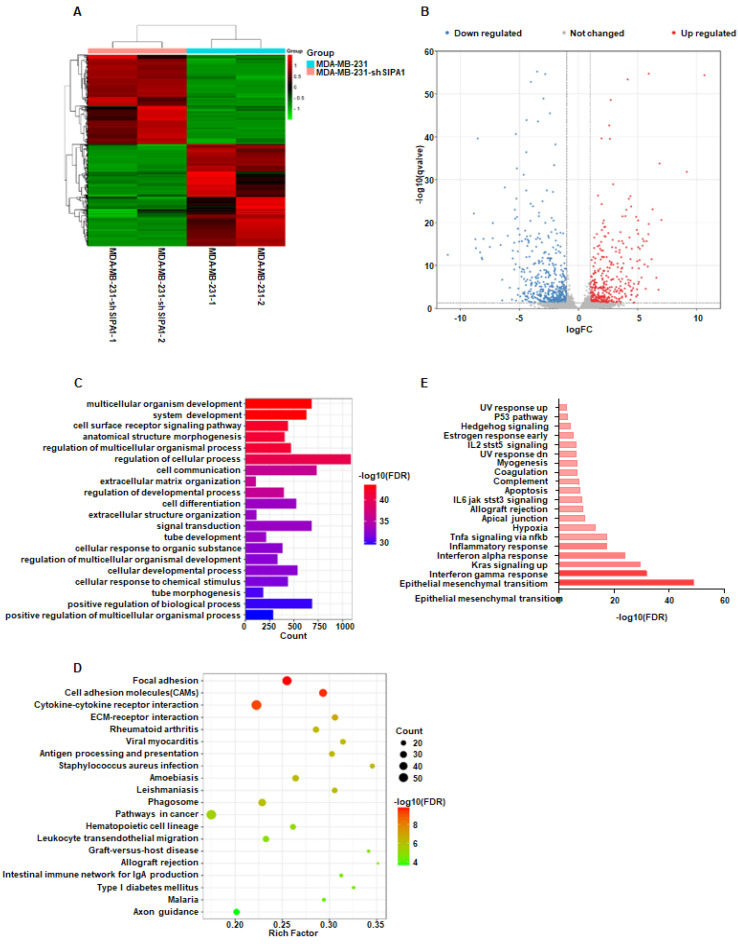
SIPA1 regulates LINC01615 expression. (**A**) Visual heat map of differential lncRNAs. (**B**) Volcanic map of differential lncRNAs. (**C**) Visual bar chart of GO enrichment biological process entries for differential lncRNA co-expression of targeted mRNAs. (**D**) Visualized bubble map of KEGG pathway of differential lncRNAs co-expressing targeted mRNAs. (**E**) Visualized bars of MsigDB landmark genome contrast of different lncRNAs co-expressing targeted mRNAs.

Previous studies have shown that *SIPA1* can promote metastasis by regulating adhesion, drug resistance, and stem ability of breast cancer cells. Other research showed that lncRNAs can regulate tumor cell metastasis, but the mechanism of whether *SIPA1* can regulate lncRNA to promote metastasis of breast cancer cells remains unclear. To understand whether SIPA1 can promote breast cancer cell metastasis by regulating the lncRNA expression in breast cancer cells, we performed high-throughput sequencing on MDA-MB-231 cells and MDA-MB-231 cells with stable knockdown of SIPA1 (Figure S1). Through differential analysis of sequencing data, we found that 385 overexpressed lncRNAs and 468 were down-regulated (Figure 2A,B). The results of GO analysis, KEGG analysis, and MsigDB gene set alignment analysis showed that the biological function of differential lncRNAs might be related to cancer cell metastasis, and the comparison results with MsigDB marker genome suggested that differential lncRNAs might be related to cell EMT process (Figure 2C–E). Suggesting that SIPA1 may regulate breast cancer cell metastasis by regulating the expression of this lncRNA and then the EMT process. According to the expression level of differential lncRNAs and the correlation between differential lncRNAs and migration function, six candidate lncRNAs were screened out: MIR100HG, LINC00702, LINC01615, LINC02544, Sertad4-AS1, and FENDRR for subsequent experiments. The expression levels of candidate lncRNAs in breast cancer cell lines were verified by RT-qPCR, and only LINC01615 was found to meet expectations (Figure S2). The gene expression data and clinical information of breast cancer tissue samples were downloaded from TCGA database. The samples were divided into high and low expression group according to the differential lncRNA expression level, and the survival prognosis discrepancy of patients in these groups was analyzed for candidate lncRNA. Only LINC01615 was significantly correlated with the survival prognosis of breast cancer patients, and the prognosis of patients with upregulated LINC01615 expression was significantly lower than that with low LINC01615 expression (Figure S3). At the same time, the breast cancer gene expression samples were downloaded from the TCGA database and analyzed the candidate lncRNAs expression in tumor tissues (*n* = 1090) and normal adjacent tissues (*n* = 113). The results showed that the expression level of LINC01615 in tumor tissues was higher than that in normal breast tissues (Figure S4).

### 3.3. LINC01615 Promotes Breast Cancer Cells Migration and Invasion

**Figure 3:**
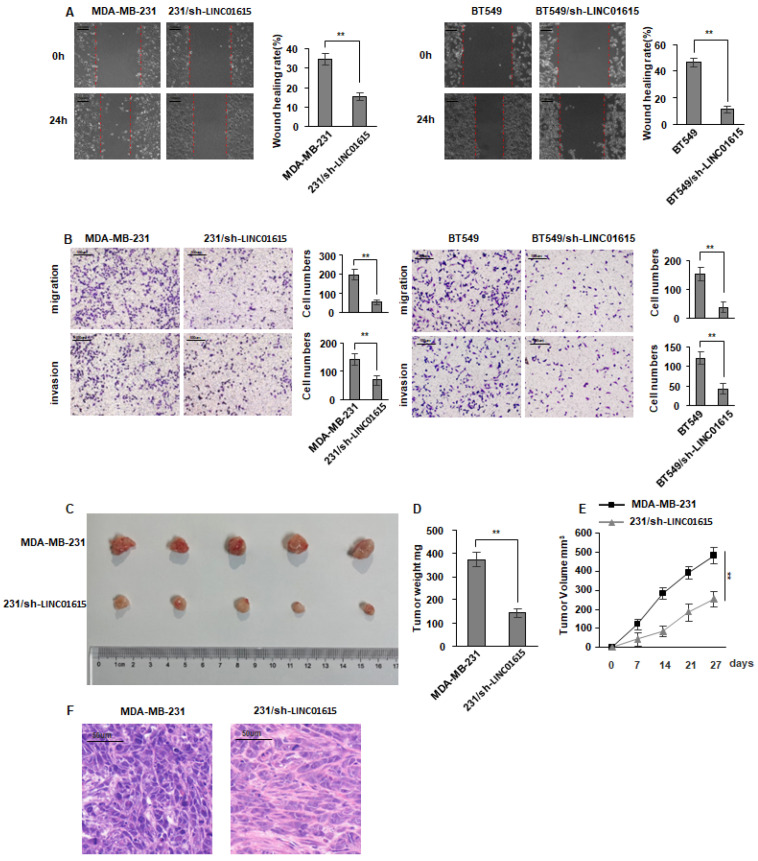
LINC01615 promotes migration and invasion of breast cancer cells. (**A**,**B**) BT549 and MDA-MB-231 cells were transduced with shLINC01615 or shNC as indicated. Cell metastasis was determined by Scratch wound assays (**A**) or Transwell migration and Matrigel invasion assays (**B**). (**C**,**D**) Subcutaneous xenografts of breast cancer cells infected with LINC01615 knockdown lentivirus (sh) or control lentivirus (shNC). (**C**) Images of the tumors at autopsy from nude mice are presented. (**D**,**E**) Tumor weight and volume of xenografted tumors were measured, respectively. (**F**) Representative H&E-stained sections of the tumor tissues isolated from mice. Data represent the means ± SD. ** *p* < 0.01.

Furthermore, the LINC01615 knocked down in BT549 and MDA-MB-231 cells were made. Performing that, the cell migration and invasion were suppressed (Figure 3A,B). Meanwhile, stably knocked down LINC01615 MDA-MB-231 cells were constructed, and 5×10^6^ cells were injected into 4-week-old female nude mice subcutaneously. Tumor volumes were measured weekly. After 28 days, all mice were euthanized for tumor dissection. The tumor samples were then photographed for measurement. The result showed that the tumorigenic ability of LINC01615 knockdown MDA-MB-231 cells was significantly reduced, as well as the tumor volume and weight (Figure 3C–F). This further indicates that LINC01615 can promote breast cancer cell migration and invasion.

### 3.4. Overexpression of LINC01615 Could Counteract the Inhibition of SIPA1 Knockdown

**Figure 4:**
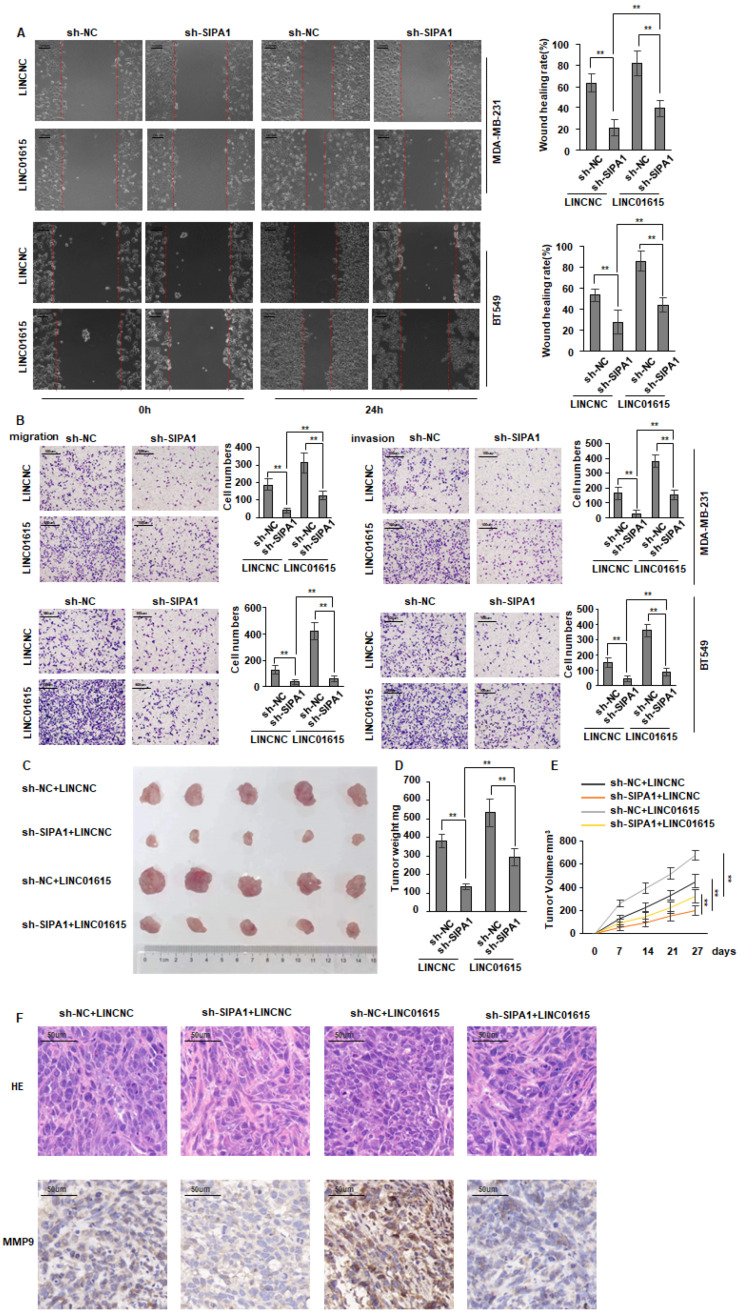
Overexpression of LINC01615 could counteract the inhibition of SIPA1 knockdown. (**A**,**B**) BT549 and MDA-MB-231 cells were transfected with control LINC, LINC01615, shRNA, and SIPA1 shRNA as indicated. (**A**) Scratch wound assays and (**B**) Transwell migration and Matrigel invasion assays were performed 24 h after transfection. (**C**,**D**) Subcutaneous xenografts of MDA-MB-231 cells infected with LINC01615 lentivirus and SIPA1 knockdown lentivirus (shSIPA1). (**C**) Images of the tumors at autopsy from nude mice are presented. (**D**,**E**) Tumor volume and weight of xenografted tumors were measured. (**F**) Representative H&E-stained sections and Immunohistochemical analysis of the tumor tissues isolated from mice. Data represent the means ± SD. ** *p* < 0.01.

To further verify that SIPA1 promotes the malignancy of breast cancer by inducing LINC01615 expression, we knocked down SIPA1 in BT549 and MDA-MB-231 cells and simultaneously overexpressed LINC01615. Indicating that overexpression of LINC01615 could counteract cell migration and invasion inhibited by SIPA1 knockdown (Figure 4A,B). We constructed MDA-MB-231 cells with simultaneous knockdown of SIPA1 and overexpression of LINC01615, and 5 × 10^6^ cells were subcutaneously injected into 4-week-old female nude mice. After 28 days, all mice were euthanized for tumor dissection. We found that the decreased tumor volume caused by SIPA1 knockdown was offset by overexpression of LINC01615 (Figure 4C–F). These data fully proved that LINC01615 was subjected to SIPA1.

### 3.5. LINC01615 Promotes the Expression of MMP9

**Figure 5:**
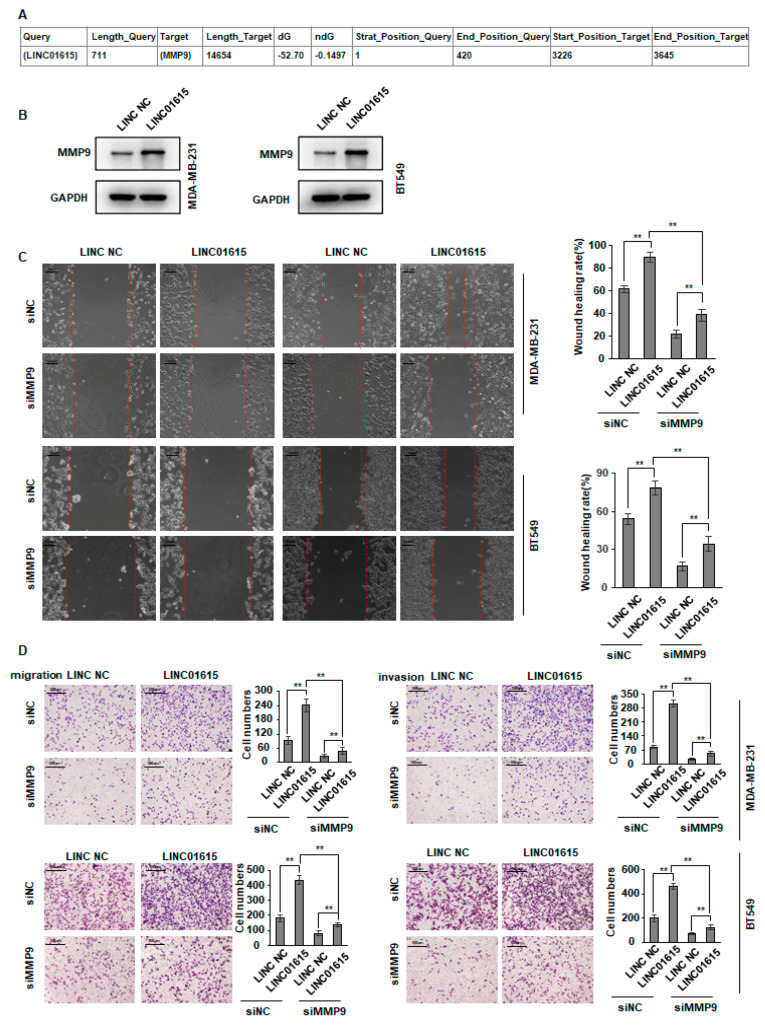
LINC01615 promotes the expression of MMP9. (**A**) Prediction of LINC01615 binding site to MMP9. (**B**)The expression of MMP9 was detected by western blot (the original western blot can be found in Figure S5). (**C**,**D**) BT549 and MDA-MB-231 cells were transduced with LINC01615 or siMMP9 as indicated. Cell metastasis was determined by Scratch wound assays (**C**) or Transwell migration and Matrigel invasion assays (**D**). ** *p* < 0.01.

Through bioinformatics website analysis, it was found that LINC01615 could bind to MMP9 to stabilize its structure (Figure 5A). After overexpression of LNC01619, we found that the expression of MMP9 was also increased (Figure 5B).To further verify this hypothesis, we knocked down MMP9 after overexpression of LINC01615 in BT549 and MDA-MB-231 cells and found that migration and invasion induced by overexpression of LINC01615 were inhibited by knockdown MMP9 (Figure 5C,D). These results further confirm the molecular mechanism by which SIPA1 and LINC01615 promote migration and invasion.

## 4. Discussion

In clinical practice, surgery is the first choice for breast cancer treatment, and endocrine therapy and chemoradiotherapy are selected according to individual differences. Clinically, there is an urgent need for new diagnostic methods and treatment programs so as to improve the early detection rate of breast cancer, reduce the treatment damage and side effects as much as possible, and achieve “precision treatment” has become the research direction of the majority of scholars [1,26]. In recent years, more and more scholars have focused on cancer-related molecular markers and gene targets, such as PAK1 and SOX8 [27,28]. At present, a variety of breast-cancer-related targets and targeted drugs have emerged, which provide a new means for the early diagnosis and treatment of breast cancer. Our study clarified that LINC01615 can be used as an oncogenic factor to promote the development of breast cancer, which will provide new ideas for the diagnosis and treatment of breast cancer.

SIPA1 has been studied for nearly 30 years since it was first discovered in 1995. In almost all tumor types studied, the expression level of SIPA1 is correlated with lymph node metastasis, indicating that SIPA1 may be a molecule related to tumor development and patient prognosis [17,19]. Whether SIPA1 can be used as a reliable prognostic marker molecule or even a tumor therapeutic target still needs more laboratory and clinical studies. Studies have reported that the SIPA1 protein can accelerate the development of tumors by regulating the adhesion and infiltration of various tumor cells [29,30]. In this study, overexpressed SIPA1 was found in breast cancer tissues by pan-cancer analysis, and the migration assay and fluorescence quantitative PCR assay were used to verify that the SIPA1 protein could promote the EMT process of breast cancer cells to regulate metastasis. In order to determine whether SIPA1 can affect metastasis by regulating the EMT process through lncRNA, high-throughput RNA sequencing was performed on MDA-MB-231 and MDA-MB-231/SH-SIPA1 breast cancer cells with knockdown of SIPA1 expression level, and RNA expression data of the two cells were obtained. Subsequently, the data were filtered for quality control, and the differential expression analysis was performed to screen out the lncRNAs with significant differences, suggesting that SIPA1 could regulate the expression of lncRNAs. Subsequently, the effect of LINC01615 on the metastasis of triple-negative breast cancer cells was studied in MDA-MB-231 cells. Through scratch test and Transwell invasion test, it was proved that overexpression of LINC01615 expression could promote the migration and invasion ability of breast cancer cells. Then the effect of LINC01615 on regulating MMP9 was performed by western blot. The results indicated that the high expression of LINC01615 could promote the expression of MMP9 in breast cancer.

LncRNAs are involved in various cellular functions and physiological processes in the nucleus and cytoplasm [31,32]. In the nucleus, lncRNAs can be involved in chromatin remodeling, chromatin modification, or pre-transcriptional gene expression regulation. In the cytoplasm, lncRNAs are mainly involved in post-transcriptional regulation and post-transcriptional modification [33,34]. At present, studies on the biological functions of lncRNA in breast cancer mainly focus on the influence of lncRNA on drug resistance, apoptosis, proliferation, and metastasis of breast cancer cells, etc. However, there are few reports on the expression of lncRNA and its biological role in triple-negative breast cancer [35,36]. This study explored the expression of lncRNA regulated by SIPA1 protein in triple-negative breast cancer cells and studied its biological function, which is of great significance for exploring the potential diagnostic and therapeutic value of lncRNA in breast cancer and promoting the treatment of tumor metastasis in triple-negative breast cancer patients. LINC01615 can potentially become a target molecule in the cancer metastasis therapy of breast cancer patients. Previous studies have shown that LINC01615 promotes tumorigenesis and progression in hepatocellular carcinoma, colon cancer, and clear cell renal cell carcinoma [37,38,39]. Moreover, the expression of LINC01615 is elevated in breast cancer patients, and overexpressed LINC01615 was associated with a poor prognosis. Further studies proved that LINC01615 could promote malignancy in breast cancer cells. Western blot results showed that LINC01615 could promote the expression of migration marker protein MMP9.

Previous studies have shown that the interaction between SIPA1 and several molecules is known to regulate cell proliferation and migration and can provide potential models for studying the process of tumor metastasis, such as BRD4. More and more evidence shows that SIPA1 not only regulates tumor occurrence and development through intermolecular interaction but also regulates gene transcription in the nucleus. Known target genes include CD44 and ITGB1, and SIPA1 protein can regulate Smad2/3 molecular mRNA level [24,40]. This study shows that SIPA1 regulates one of the downstream pathways of breast cancer metastasis; namely, SIPA1 can regulate the expression of the corresponding lncRNA to regulate metastasis. However, how SIPA1 regulates the expression of lncRNA needs to be further studied. Subsequent studies can be carried out by analyzing the sequence of SIPA1 and lncRNA and proving whether SIPA1 can directly bind to lncRNA. On the other hand, the role of lncRNA in cell proliferation was only preliminarily studied in this study, and the role of lncRNA in cell proliferation and autophagy could be further studied in the future. Later, the downstream mechanism of lncRNA can be further studied.

In summary, we confirmed the regulatory effect of SIPA1 on LINC01615 in breast cancer cell lines, which is ultimately reflected in cell migration and invasion and tumor metastasis in vivo. This study provides a better understanding of the molecular mechanisms behind breast cancer metastasis, which is a major cause of poor prognosis. Therefore, clarifying the specific biological mechanism of this phenomenon can better diagnose and treat the disease process.

## 5. Conclusions

In conclusion, our study found that the up-regulation of SIPA1 can promote the expression of LINC01615, and the up-regulation of LINC01615 can promote the expression of MMP9 to promote the migration and invasion of triple-negative breast cancer. Our experimental results will provide a new theoretical basis for the diagnosis and treatment of triple-negative breast cancer.

## Data Availability

The data presented in this study are available in this article (and Supplementary Material).

## Supplementary Materials

The following supporting information can be downloaded at: https://www.mdpi.com/article/10.3390/cancers14194815/s1, Figure S1: High-throughput sequencing data output and post-QC results; Figure S2: The expression of LINC01615 was decreased after knockdown of SIPA1; Figure S3: The expression level of LINC01615 was negatively correlated with the prognosis of breast cancer. Figure S4: LINC01615 expression is elevated in breast cancer tissues. Figure S5: Original Western Blot.
