# Supplementary material for: SIPA1 Regulates LINC01615 to Promote Metastasis in Triple-Negative Breast Cancer
Source: Cancers (Basel). 2022 Oct 1;14(19):4815. doi: 10.3390/cancers14194815 (PMC9562673; doi:10.3390/cancers14194815)
Supplement: Supplementary file 1 [file cancers-14-04815-s001.zip › cancers-1930082-supplementary.pdf]

Supplementary Materials

SIPA1 Regulates LINC01615 to Promote Metastasis in Triple-Negative Breast Cancer

Yuan Xiang, Lingyun Feng, Hui Liu, Yuhuan Liu, Jiapeng Li, Li Su and Xinghua Liao

Sample	Total Raw Reads (M)	Total Clean Reads (M)	Total Clean Bases (Gb)	Clean Reads Q30 (%)	Clean Reads Ratio (%)
MDA-MB-231_1	119.94	113.48	11.35	92.83	94.62
MDA-MB-231_2	119.94	113.12	11.31	93.28	94.32
MDA-MB-231/sh-SIPA1_1	119.94	113.71	11.37	93.36	94.81
MDA-MB-231/sh-SIPA1_2	119.94	114.03	11.4	93.77	95.08

Figure S1. High-throughput sequencing data output and post-QC results.

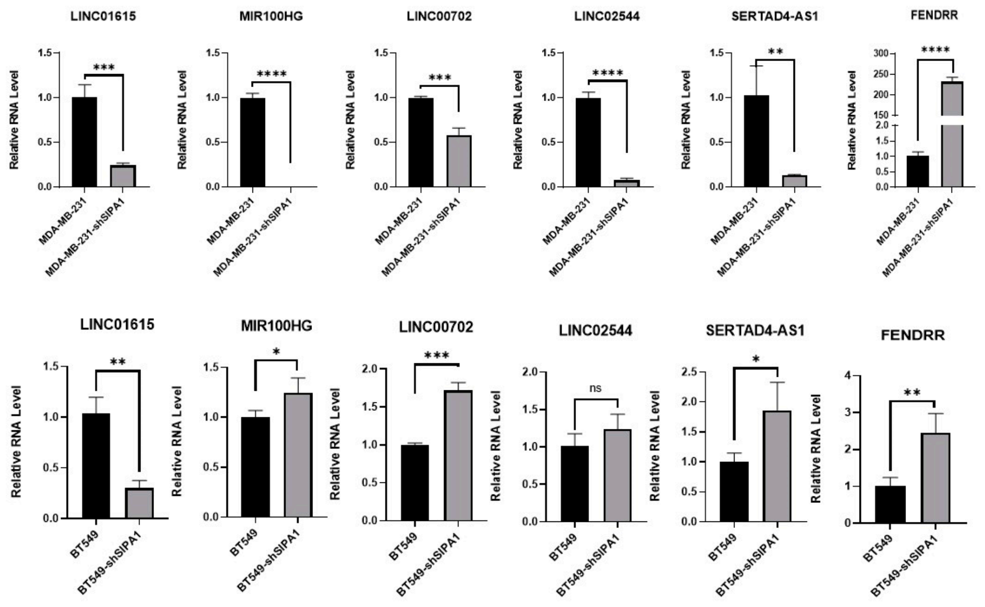

Figure S2. The expression of LINC01615 was decreased after knockdown of SIPA1. Real-time RT-PCR was used to detect the relative expression of MIR100HG, LINC00702, LINC01615, LINC02544, SERTAD4-AS1 and FENDRR in BT549 and BT549/sh-SIPA1 and MDA-MB-231 and MDA-MB-231/sh-SIPA1 cells. Data represent the means ± SD. **P < 0.01.

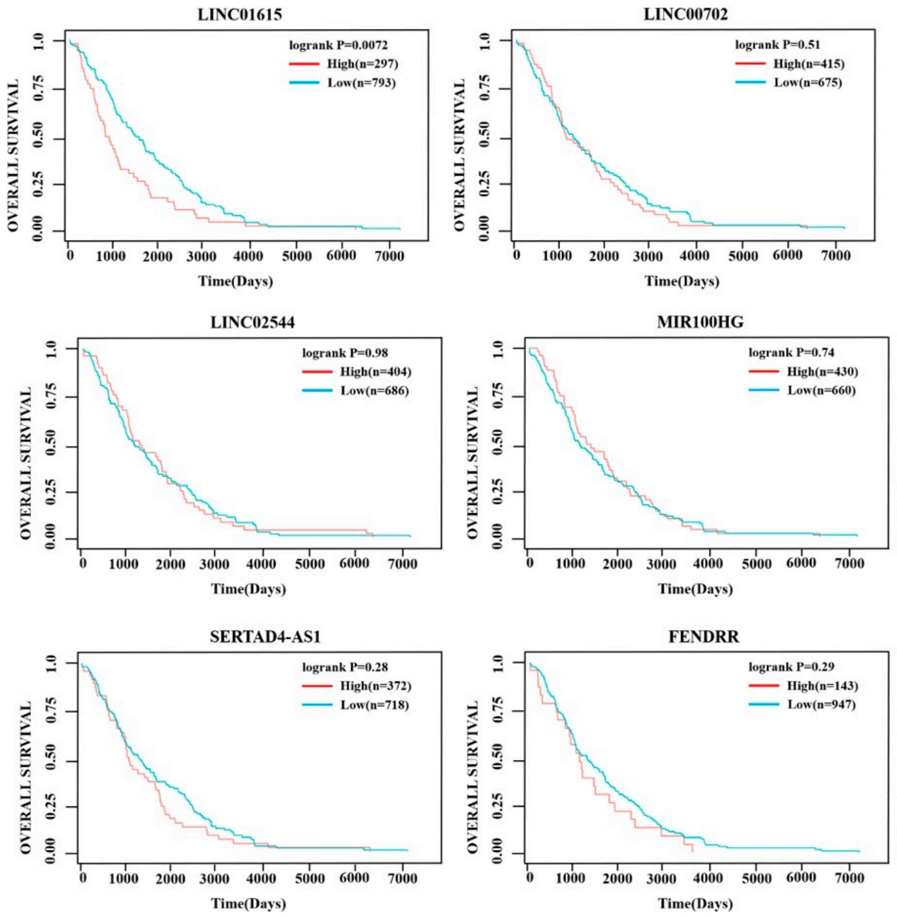

Figure S3. The expression level of LINC01615 was negatively correlated with the prognosis of breast cancer. Survival analysis of MIR100HG, LINC00702, LINC01615, LINC02544, SERTAD4-AS1 and FENDRR in breast cancer patients.

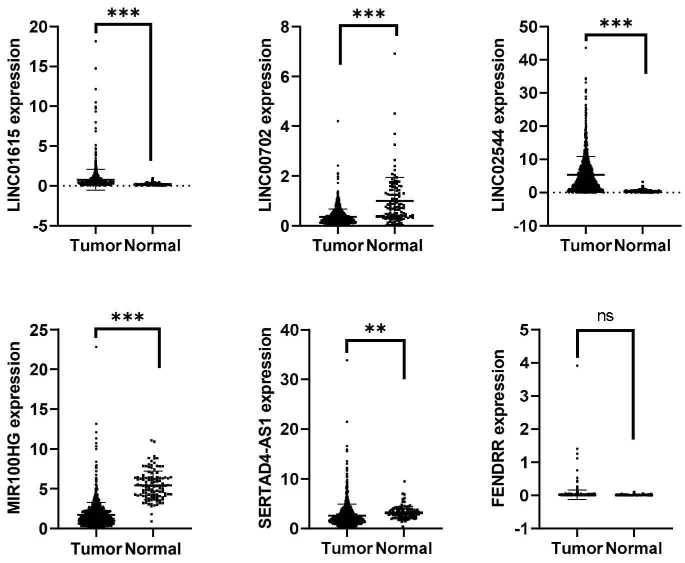

Figure S4. LINC01615 expression is elevated in breast cancer tissues. Differences in the expression levels of MIR100HG, LINC00702, LINC01615, LINC02544, SERTAD4-AS1 and FENDRR in breast tumor tissues and normal breast tissues.
